# Foundational Basis for the Development of MEDSReM

**DOI:** 10.1093/geroni/igab046.880

**Published:** 2021-12-17

**Authors:** Kathleen Insel, Gilles Einstein, Daniel Morrow, Jeannie Lee, Wendy Rogers, Tracy Mitzner

**Affiliations:** 1 University of Arizona, Tucson, Arizona, United States; 2 Furman University, Greenville, South Carolina, United States; 3 University of Illinois Urbana-Champaign, Champaign, Illinois, United States; 4 Georgia Institute of technology, Atlanta, Georgia, United States

## Abstract

Discovering a composite of measures of executive function/working memory predicted everyday medication adherence among older adults, led to the development of a behavioral intervention, the Multifaceted Prospective Memory Intervention (MPMI) to improve hypertension medication adherence. The intervention resulted in a 35% improvement in adherence compared to an active education and attention control condition. However, adherence slowly declined over an additional five months of adherence monitoring without the presence of interventionists in the home. We proposed that the use of technology might help individuals maintain the prospective memory strategies, resulting in sustained adherence. An interdisciplinary team was formed to translate the behavioral intervention to technology, resulting in the first version of the MEDSReM system. In this presentation we describe the evolution of the project, from the components of the successful MPMI to the design and initial testing of MEDSReM. These efforts provide general insights about translating interventions into technology tools.

